# Supplementary material for: Epigenetic Modulation of Radiation-Induced Diacylglycerol Kinase Alpha Expression Prevents Pro-Fibrotic Fibroblast Response
Source: Cancers (Basel). 2021 May 18;13(10):2455. doi: 10.3390/cancers13102455 (PMC8158145; doi:10.3390/cancers13102455)

Figure S1

A

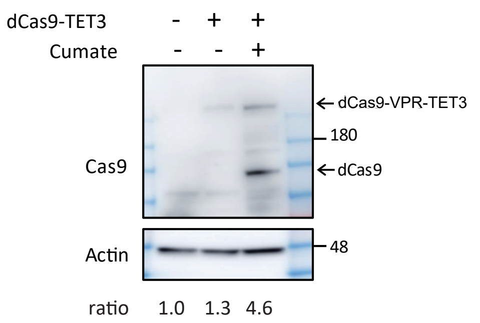

B

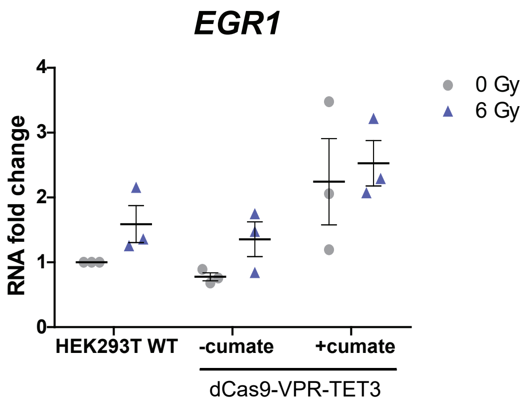

C

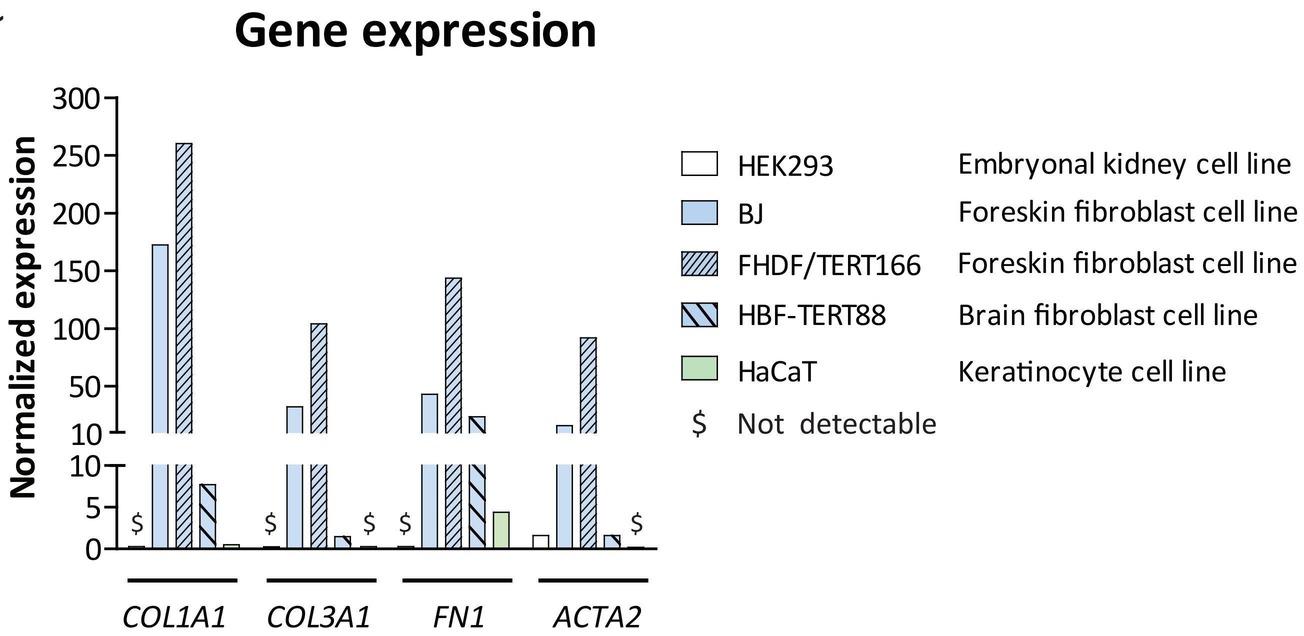

Figure S1. dCas9-VPR-TET3 expression and endogenous relative mRNA expression of indicated genes in human cell lines

(A) Protein expression of Cas9 in dCas9-VPR-TET3-transfected HEK293T cells. Cells were treated with or without cumate (300 μg/ml) for dCas9-VPR-TET3 induction over 2 passages before harvesting. (B) *EGR1* expression in HEK293T and dCas9-VPR-TET3-transfected cells treated without or with cumate (300 μg/ml). Cells were harvested 48 hours after irradiation with 6 Gy. Data include three biological replicates and are presented as mean± SEM. Statistical significance was determined by one-tailed Student's t test. (C) Endogenous relative mRNA expression of indicated genes in human cell lines. Data are derived from a consensus dataset in the Human Protein Atlas (HPA), which combines data from three comprehensive databases (HPA, GTEx, and FANTOM5 [52]).

Figure S2

A

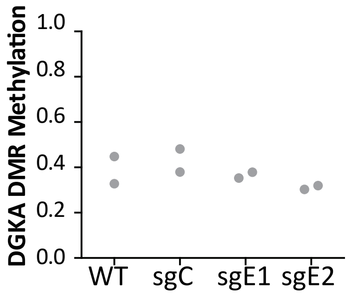

B

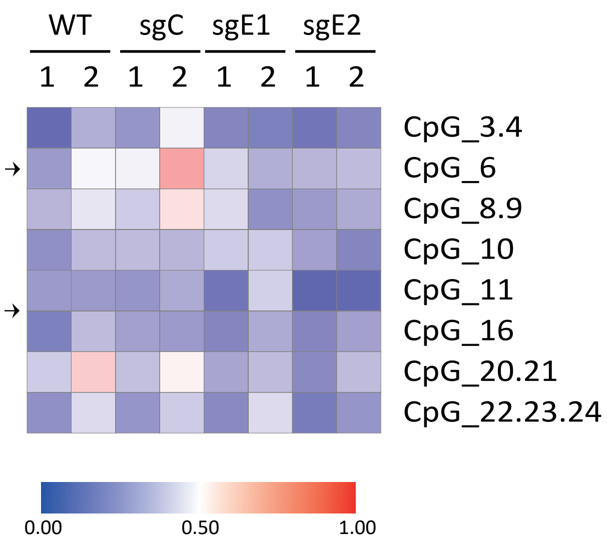

C

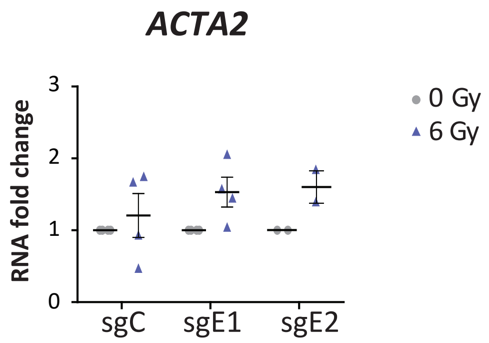

D

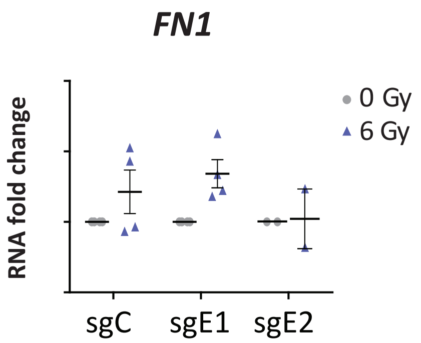

Figure S2. BJ cells with CRISPR/Cas9-edited EGR1-binding sites at the *DGKA* enhancer

(A) Methylation average across various CpGs located in the *DGKA* enhancer in parental (WT) and CRISPR/Cas9-edited (sgC, sgE1 and sgE2) BJ cells. Methylation was measured as β-values by EpiTYPER technology. (B) Heatmap of DNA methylation of all CpG sites measured in parental (WT) and CRISPR/Cas9-edited (sgC, sgE1 and sgE2) BJ cells. For each cell type, two replicates (1,2) are shown. Arrows indicate the location of the EGR1-binding motifs. The first EGR1-binding site (EGR1_1) covers CpG_6, and the second binding site (EGR1_2) covers CpG_14, which is located between CpG_11 and CpG_16 and not detected by EpiTYPER. For (A and B), data are depicted from duplicate experiments in each cell line. (C-D) Relative mRNA expression of *ACTA2* (C) and *FN1* (D) in CRISPR/Cas9-edited (sgC, sgE1 and sgE2) BJ cells. Cells were harvested 48 hours after irradiation with 6 Gy. For (C and D), data include at least two biological replicates and are presented as mean± SEM. Statistical significance was determined by one-tailed Student's t test.

Figure S3

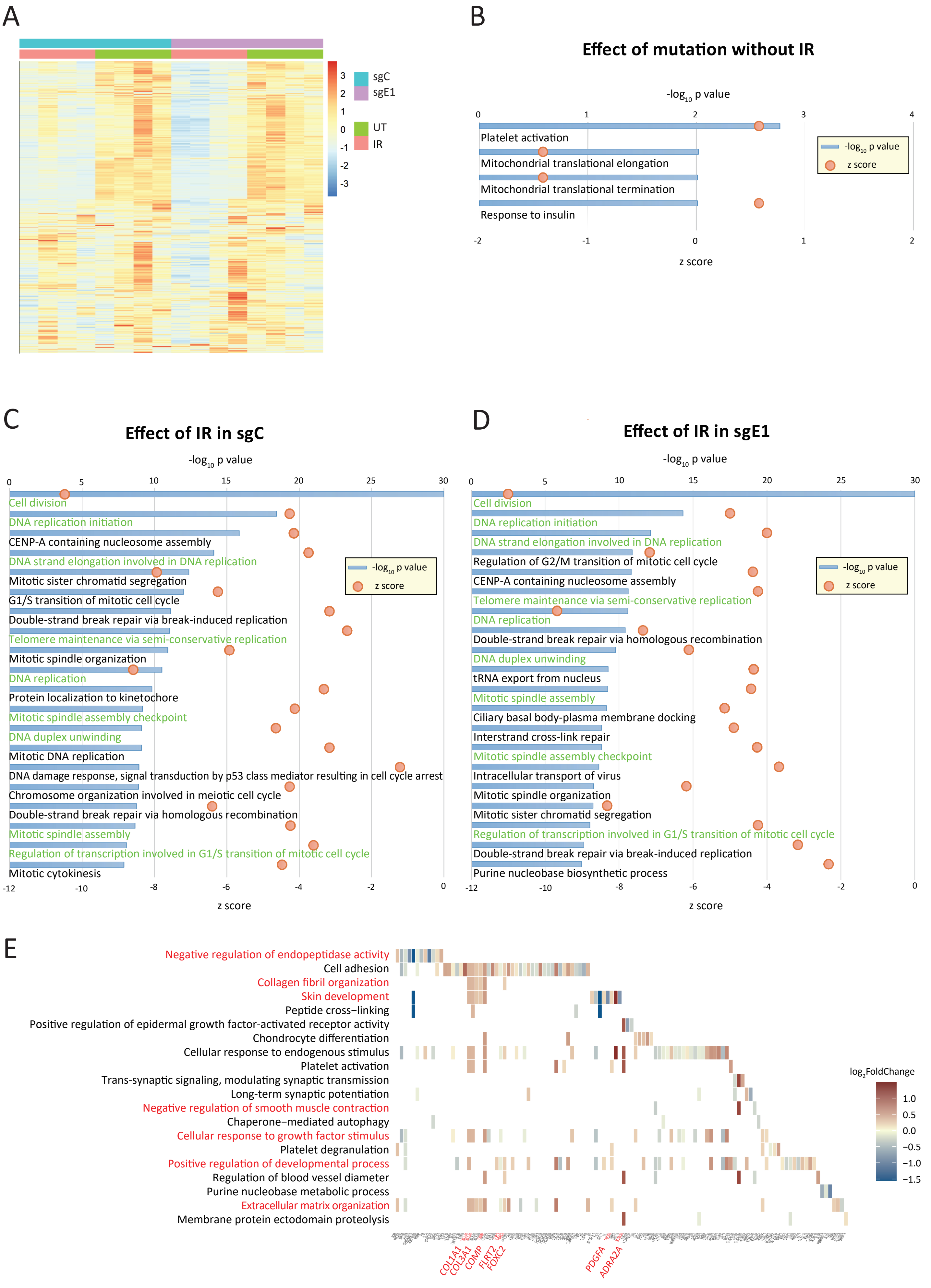
A
sgC
sgE1
UT
IR
3
2
1
0
-1
-2
-3
B
Effect of mutation without IR
-log10 p value
Platelet activation
Mitochondrial translational elongation
Mitochondrial translational termination
Response to insulin
z score
C
Effect of IR in sgC
-log10 p value
Cell division
DNA replication initiation
CENP-A containing nucleosome assembly
DNA strand elongation involved in DNA replication
Mitotic sister chromatid segregation
G1/S transition of mitotic cell cycle
Double-strand break repair via break-induced replication
Telomere maintenance via semi-conservative replication
Mitotic spindle organization
DNA replication
Protein localization to kinetochore
Mitotic spindle assembly checkpoint
DNA duplex unwinding
Mitotic DNA replication
DNA damage response, signal transduction by p53 class mediator resulting in cell cycle arrest
Chromosome organization involved in meiotic cell cycle
Double-strand break repair via homologous recombination
Mitotic spindle assembly
Regulation of transcription involved in G1/S transition of mitotic cell cycle
Mitotic cytokinesis
z score
D
Effect of IR in sgE1
-log10 p value
Cell division
DNA replication initiation
DNA strand elongation involved in DNA replication
Regulation of G2/M transition of mitotic cell cycle
CENP-A containing nucleosome assembly
Telomere maintenance via semi-conservative replication
DNA replication
Double-strand break repair via homologous recombination
DNA duplex unwinding
tRNA export from nucleus
Mitotic spindle assembly
Ciliary basal body-plasma membrane docking
Interstrand cross-link repair
Mitotic spindle assembly checkpoint
Intracellular transport of virus
Mitotic spindle organization
Mitotic sister chromatid segregation
Regulation of transcription involved in G1/S transition of mitotic cell cycle
Double-strand break repair via break-induced replication
Purine nucleobase biosynthetic process
z score
E
Negative regulation of endopeptidase activity
Cell adhesion
Collagen fibril organization
Skin development
Peptide cross−linking
Positive regulation of epidermal growth factor-activated receptor activity
Chondrocyte differentiation
Cellular response to endogenous stimulus
Platelet activation
Trans-synaptic signaling, modulating synaptic transmission
Long-term synaptic potentiation
Negative regulation of smooth muscle contraction
Chaperone−mediated autophagy
Cellular response to growth factor stimulus
Platelet degranulation
Positive regulation of developmental process
Regulation of blood vessel diameter
Purine nucleobase metabolic process
Extracellular matrix organization
Membrane protein ectodomain proteolysis
log2FoldChange
1.0
0.5
0.0
−0.5
−1.0
−1.5
COL1A1
COL3A1
COMP
FLRT2
FOXC2
PDGFA
ADRA2A

Figure S3. Genome-wide effects induced by loss of EGR1-binding at the *DGKA* enhancer

(A) Hierarchical clustering heatmap showing all differentially expressed genes (DEGs) from all comparisons (sgC versus sgE1 cells with or without 6 Gy irradiation). (B) Gene ontology (GO) analysis of the DEGs obtained from the comparison of sgC and sgE1 cells without irradiation. (C and D) GO analysis of DEGs identified after irradiation (6 Gy) of sgC (C) and sgE1 (D) cells. Significance (-$\log_{10}$(p value)) of GO terms are represented by blue bars, and z scores are shown with red dots. The common biological functions and pathways existing in both sgC and sgE1 cells are highlighted in green. (E) A cluster heatmap of DEGs contributing to the top 20 GO pathways from the comparison of sgC and sgE1 cells after irradiation and adjustment for irradiation. Biological functions and pathways potentially related to fibroblast activation and fibrosis development are highlighted in red in (B) and (E).

Figure S4

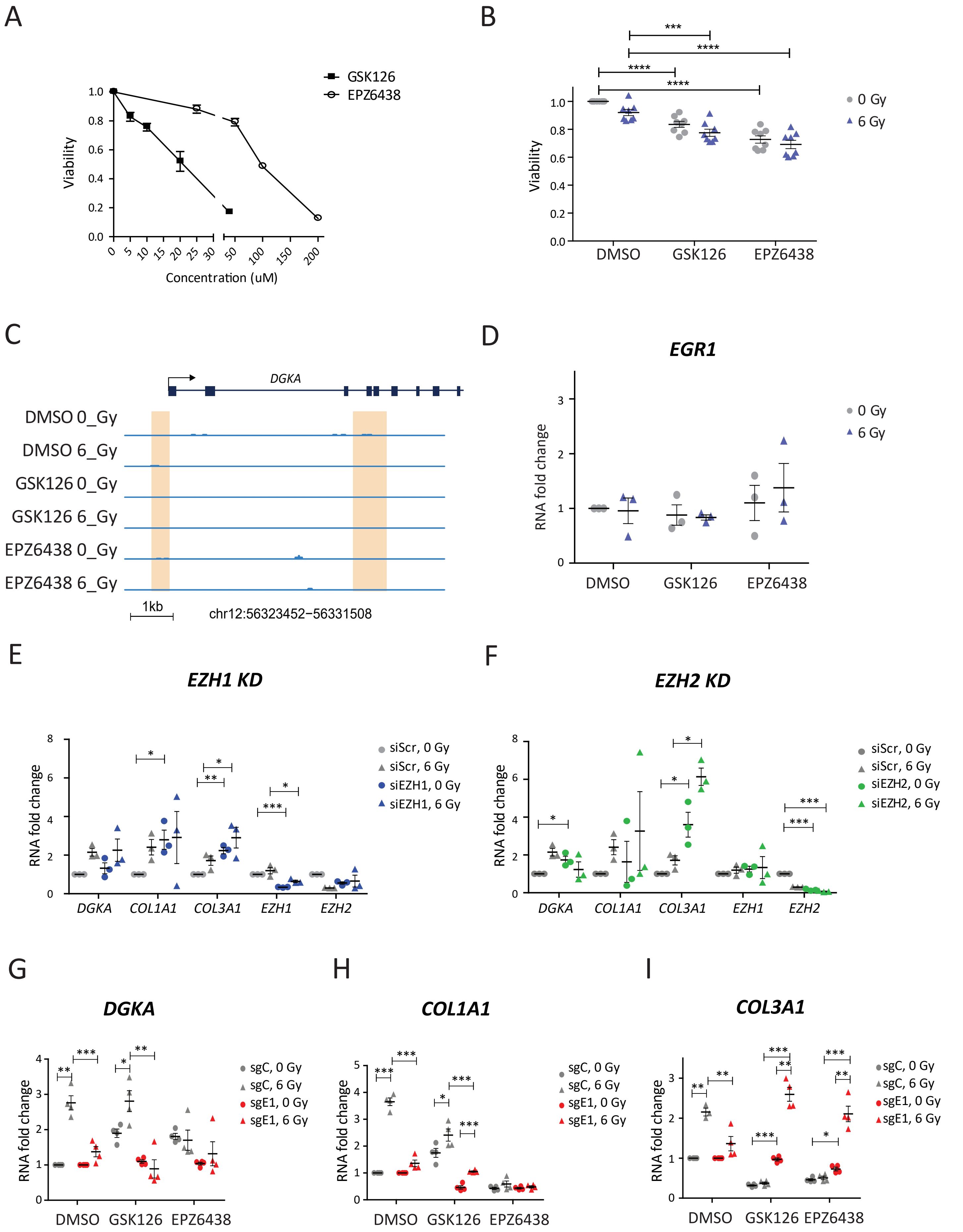
A
GSK126
EPZ6438
Viability
Concentration (uM)
B
0 Gy
6 Gy
Viability
DMSO
GSK126
EPZ6438
C
DGKA
DMSO 0_Gy
DMSO 6_Gy
GSK126 0_Gy
GSK126 6_Gy
EPZ6438 0_Gy
EPZ6438 6_Gy
1kb
chr12:56323452−56331508
D
EGR1
RNA fold change
0 Gy
6 Gy
DMSO
GSK126
EPZ6438
E
EZH1 KD
RNA fold change
siScr, 0 Gy
siScr, 6 Gy
siEZH1, 0 Gy
siEZH1, 6 Gy
DGKA
COL1A1
COL3A1
EZH1
EZH2
F
EZH2 KD
RNA fold change
siScr, 0 Gy
siScr, 6 Gy
siEZH2, 0 Gy
siEZH2, 6 Gy
DGKA
COL1A1
COL3A1
EZH1
EZH2
G
DGKA
RNA fold change
sgC, 0 Gy
sgC, 6 Gy
sgE1, 0 Gy
sgE1, 6 Gy
DMSO
GSK126
EPZ6438
H
COL1A1
RNA fold change
sgC, 0 Gy
sgC, 6 Gy
sgE1, 0 Gy
sgE1, 6 Gy
DMSO
GSK126
EPZ6438
I
COL3A1
RNA fold change
sgC, 0 Gy
sgC, 6 Gy
sgE1, 0 Gy
sgE1, 6 Gy
DMSO
GSK126
EPZ6438

Figure S4. Effects of the inhibition of EZH1 and/or EZH2 in BJ cells

(A) Cell viability of BJ cells treated with EZH2 inhibitors. Cells were treated with DMSO, GSK126 (5 µM) or EPZ6438 (50 µM) at the indicated doses for 72 hours and cell viability was measured by CellTiter Blue. (B) Cell viability after EZH2 inhibitor treatment and irradiation (6 Gy) in BJ cells. Cells were pre-treated with DMSO, GSK126 (5 µM) or EPZ6438 (50 µM) for 48 hours, irradiated with 6 Gy, and incubated for another 48 hours with concurrent inhibitor treatment before the CellTiter Blue test. (C) Genome browser tracks comparing ACT-seq signals for H3K27me3 enrichment at the *DGKA* locus after inhibitor treatments. Promoter and enhancer regions are highlighted in orange. Data present one representative replicate from Figure 4B. (D) Relative mRNA expression of *EGR1* in BJ cells treated with EZH2 inhibitors. For (C-D), cells were pre-treated with DMSO, GSK126 (5 µM) or EPZ6438 (50 µM) for 48 hours, irradiated with 6 Gy, and analysed after an additional 48 hours with concurrent drug treatment. (E and F) Relative mRNA expression levels of indicated genes in BJ cells after silencing *EZH1* (E) or *EZH2* (F), respectively. Cells were pre-treated with siRNAs for 48 hours, irradiated (6 Gy), and harvested 48 hours later. (G-I) Relative mRNA expression levels of *DGKA* (G), *COL1A1* (H) and *COL3A1* (I) in CRISPR/Cas9-edited (sgC and sgE1) BJ cells. Cells were pre-treated with DMSO, GSK126 (5 µM) or EPZ6438 (50 µM) for 48 hours, irradiated with 6 Gy, and incubated for another 48 hours with concurrent drug treatment before harvest. Data include six (A), eight (B), three (D-F) or four (G-I) biological replicates and are presented as mean± SEM. Statistical significance (* $p<0.05$ ** $p<0.01$ and ***$p<0.001$) was determined by one-tailed Student's t test.

Figure S5

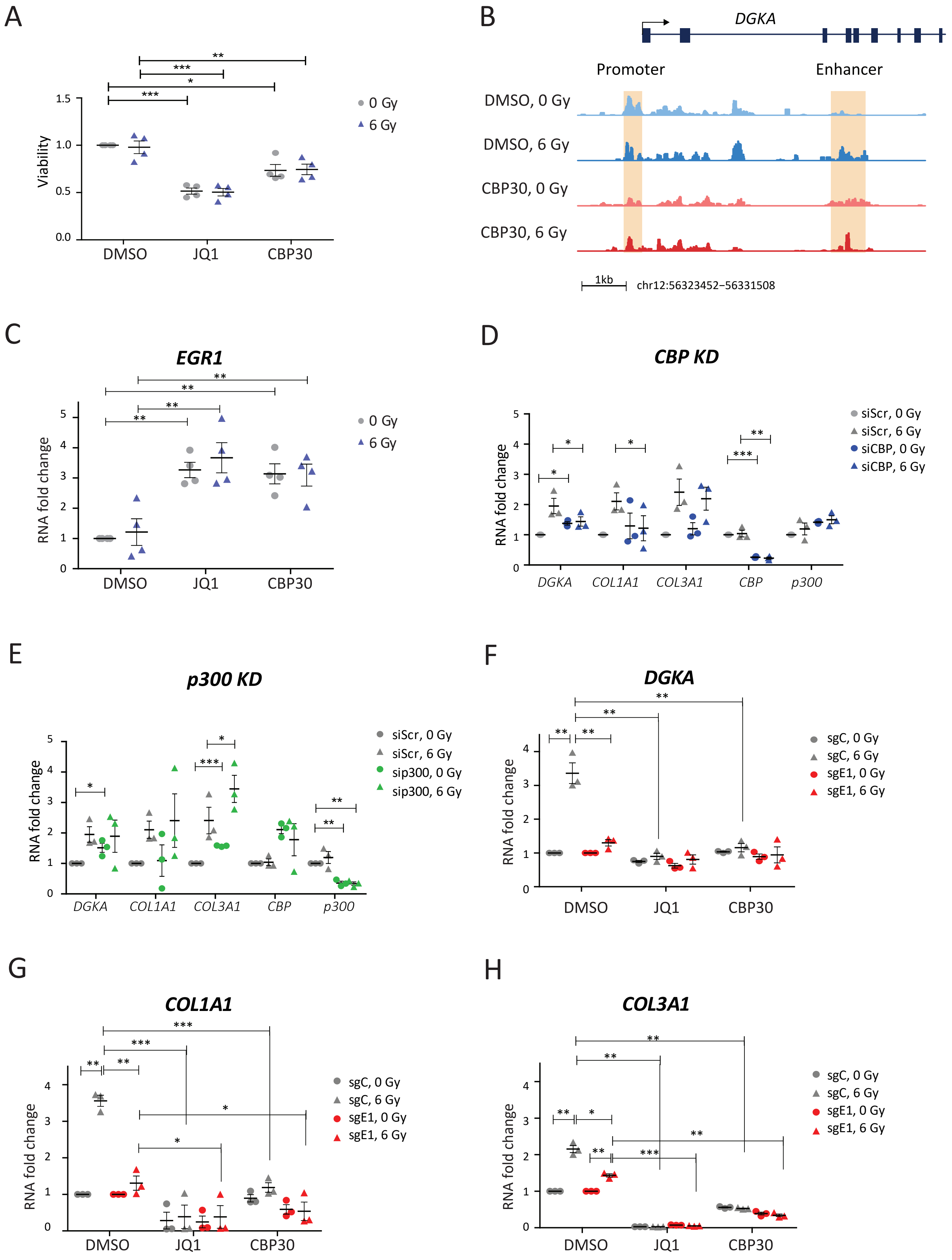
A
Viability
1.5
1.0
0.5
0.0
DMSO
JQ1
CBP30
0 Gy
6 Gy
B
DGKA
Promoter
Enhancer
DMSO, 0 Gy
DMSO, 6 Gy
CBP30, 0 Gy
CBP30, 6 Gy
1kb
chr12:56323452-56331508
C
EGR1
RNA fold change
0 Gy
6 Gy
DMSO
JQ1
CBP30
D
CBP KD
siScr, 0 Gy
siScr, 6 Gy
siCBP, 0 Gy
siCBP, 6 Gy
DGKA
COL1A1
COL3A1
CBP
p300
E
p300 KD
siScr, 0 Gy
siScr, 6 Gy
sip300, 0 Gy
sip300, 6 Gy
F
DGKA
sgC, 0 Gy
sgC, 6 Gy
sgE1, 0 Gy
sgE1, 6 Gy
G
COL1A1
H
COL3A1

Figure S5. Effects of inhibition of bromodomain proteins in BJ cells

(A) Cell viability after bromodomain inhibitor treatment and irradiation. (B) Genome browser tracks comparing ACT-seq signals for H3K27ac at the *DGKA* locus after CBP30 treatment. Promoter and enhancer regions are highlighted in orange. Data present one representative replicate from Figure 5B. (C) Relative mRNA expression of *EGR1* in BJ cells treated with bromodomain inhibitors. For (A-C), cells were pre-treated with DMSO, CBP (10 μM) or JQ1 (5 μM) for 48 hours, irradiated with 6 Gy, and incubated for another 48 hours with concurrent inhibitors before harvest. (D and E) Relative mRNA expression levels of indicated genes in BJ cells after silencing CBP (D) or p300 (E), respectively. Cells were pre-treated with siRNAs for 48 hours, irradiated with 6 Gy and harvested 48 hours later. (F-H) Relative mRNA expression levels of *DGKA* (F), *COL1A1* (G) and *COL3A1* (H) in CRISPR/Cas9-edited (sgC and sgE1) BJ cells. Cells were pre-treated with DMSO, CBP (10 μM) or JQ1 (5 μM) for 48 hours, irradiated with 6Gy, and incubated for another 48 hours with concurrent drug treatment before harvest. Statistical data are presented as mean± SEM from four (A and C) or three (D-H) biological replicates. For (B), data are presented one replicate from Figure 5B. Statistical significance (* $p<0.05$ ** $p<0.01$ and ***$p<0.001$) was determined by one-tailed Student's t test.

Figure S6

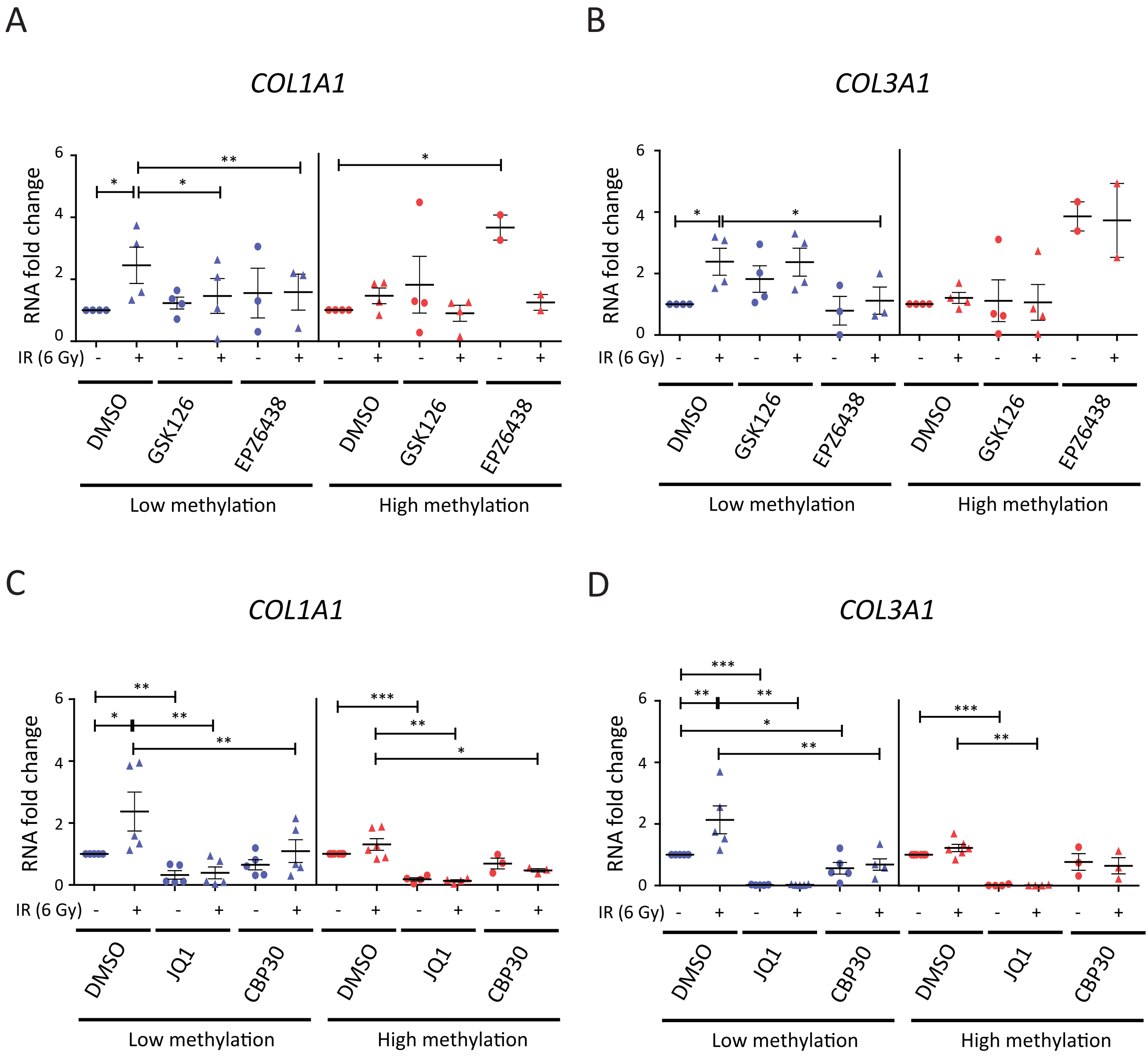

A
COL1A1
RNA fold change
6
4
2
0
IR (6 Gy)
DMSO
GSK126
EPZ6438
Low methylation
High methylation
B
COL3A1
C
COL1A1
JQ1
CBP30
D
COL3A1

Figure S6. Effects of epigenetic inhibitors in human dermal fibroblasts with low or high DNA methylation at the *DGKA* enhancer

(A-D) Relative mRNA expression of *COL1A1* (A and C) and *COL3A1* (B and D) in NHDFs. Cells were pre-treated with EZH2 inhibitors (5 µM GSK126 or 50 µM GSK126) (C and D) or bromodomain inhibitors (10 µM CBP30 or 5 µM JQ1) (E and F), irradiated with 6 Gy, and incubated for another 48 hours with concurrent drug treatment before harvest. Data points summarize effects for both fibroblast groups and include at least duplicate experiments. Data are presented as mean ± SEM, and statistical significance (* $p<0.05$ ** $p<0.01$ and ***$p<0.001$) was determined by multiple t-test.

Figure S7

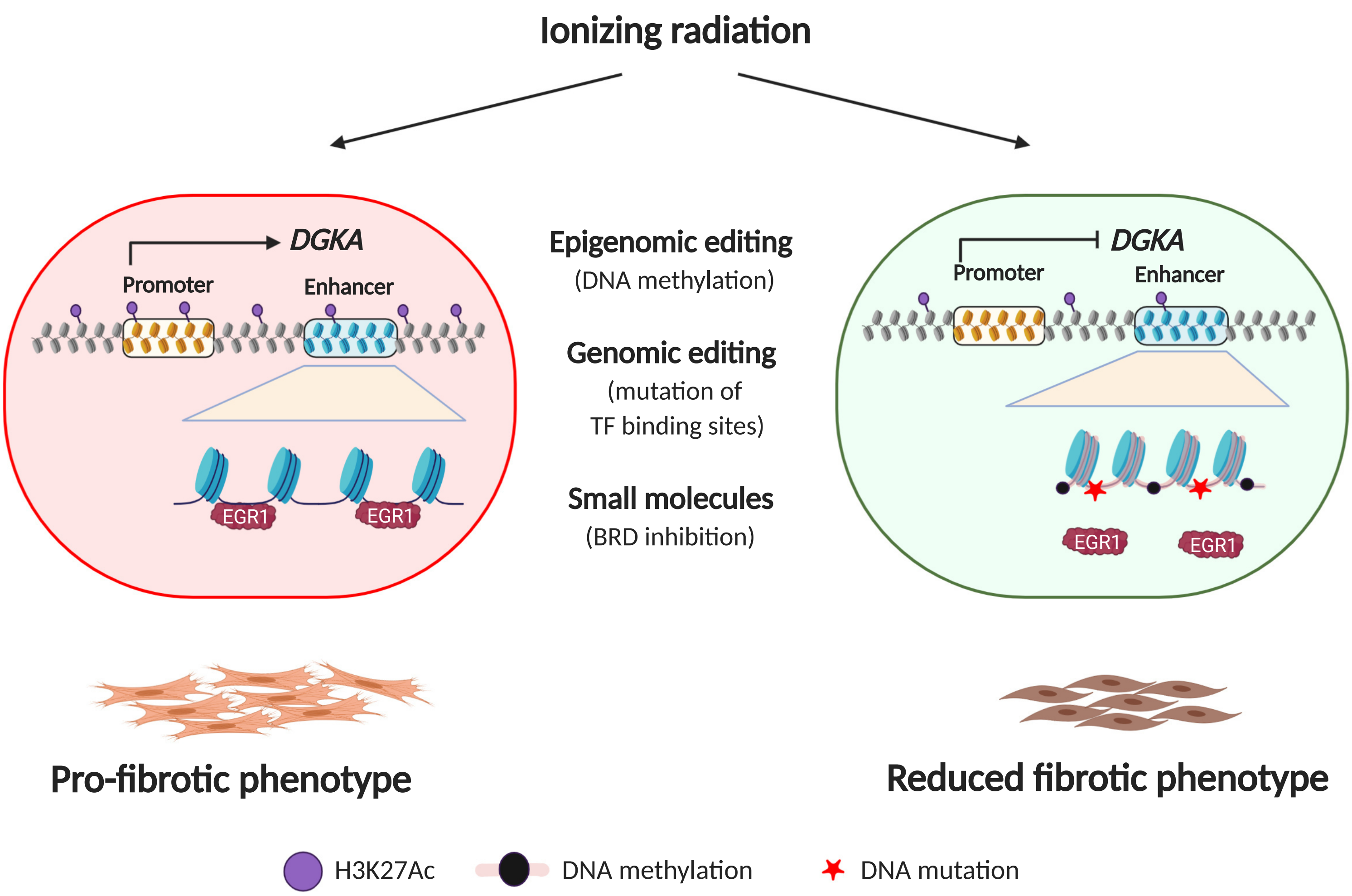

Ionizing radiation
DGKA
Promoter
Enhancer
EGR1
EGR1
Epigenomic editing
(DNA methylation)
Genomic editing
(mutation of
TF binding sites)
Small molecules
(BRD inhibition)
DGKA
Promoter
Enhancer
EGR1
EGR1
Pro-fibrotic phenotype
Reduced fibrotic phenotype
H3K27Ac
DNA methylation
DNA mutation

Figure S7. Schematic diagram of epigenetic modulation of *DGKA* expression

After ionizing radiation, unmethylated *DGKA* enhancer region enables the binding of the radiation-inducible transcription factor EGR1, thus upregulate *DGKA* pro-fibrotic marker expression. In this study, we were able to modulate radiation-induced *DGKA* expression by epigenomic (CRISPR/dCas9-VPR-TET3) and genomic (CRISPR-Cas9) editing of the *DGKA* enhancer and administering epigenetic drugs (JQ1, CBP30).

Figure S8. Original EMSA images.

Figure 1B

BTN probe E1 E1me E2 E2me

EGR1 lysate + - + - + - + -

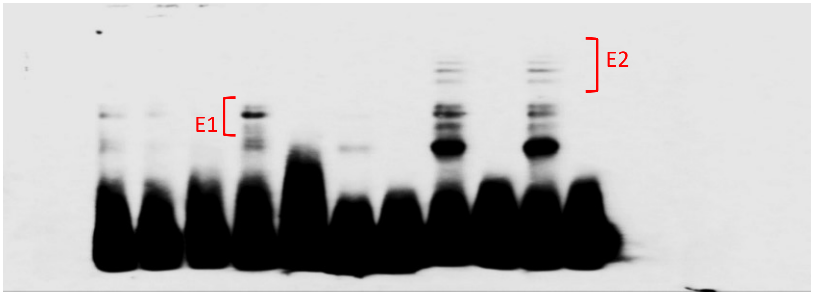

Lane 1 2 3 4 5 6 7 8

255: 31: 34: 30 | 113: 30: 127: 30

Figure 2B

BTN probe E1-WT E2-WT

EGR1 lysate - + + + + - + + +

Competitor - - E1 WT E1 mut BD1 mut - - E2 WT E2 mut

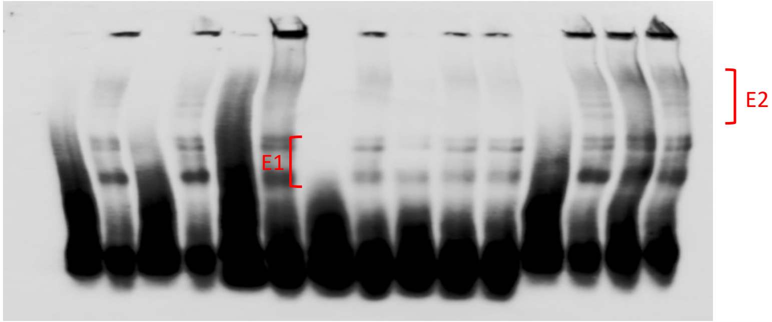

Lane 1 2 3 4 5 6 7 8 9

41: 126: 63: 110: 153| - 85: 170: 134

Figure S9. Original Western blot images for H3K27me3 and corresponding H3.

Figure 4A

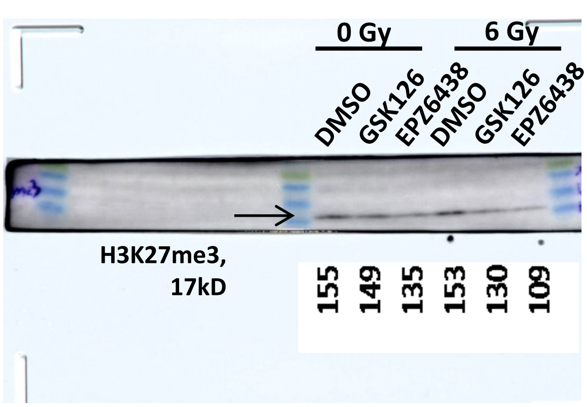

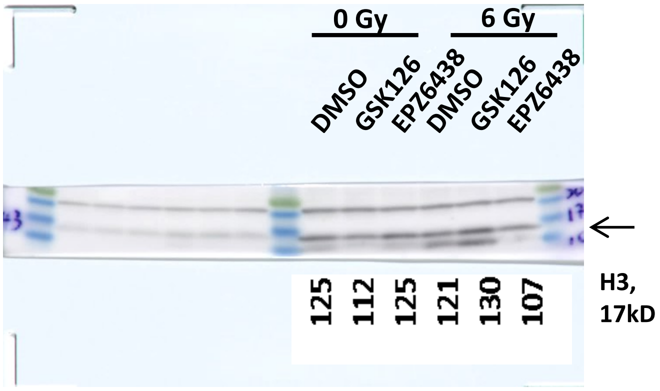

Figure 4A, image for statistic

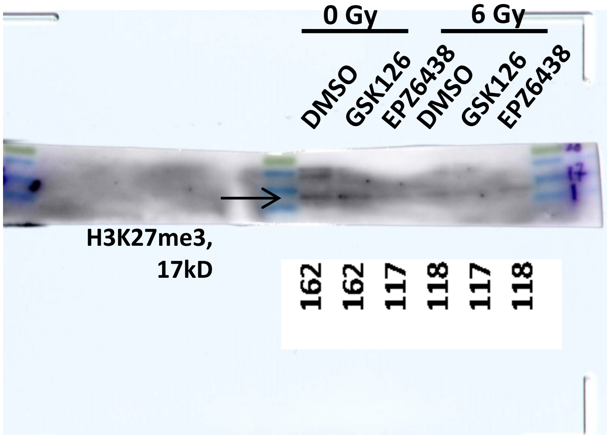

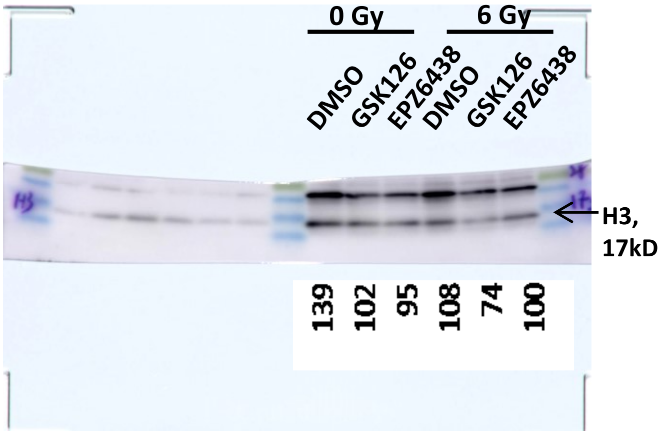

Figure S10. Original Western blot images for H3K27ac and corresponding H3.

Figure 5A

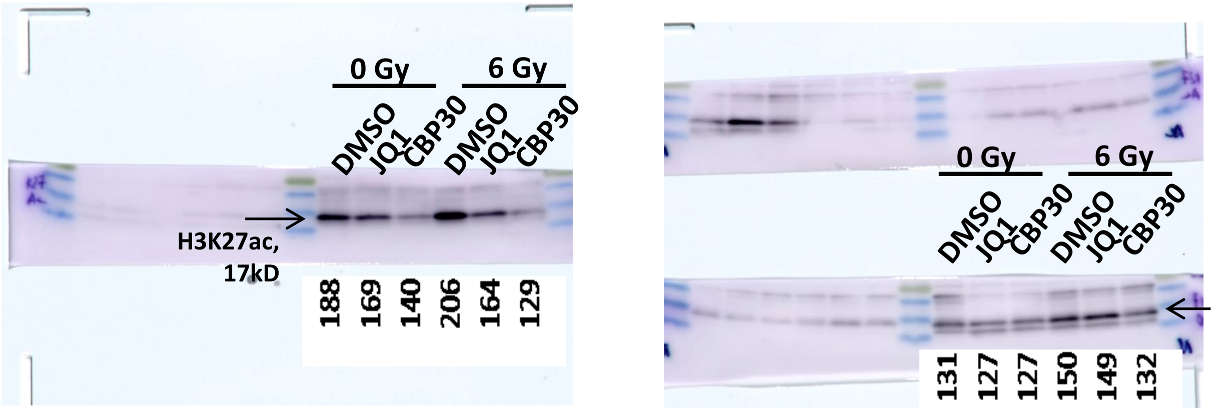

Figure 5A, image for statistic

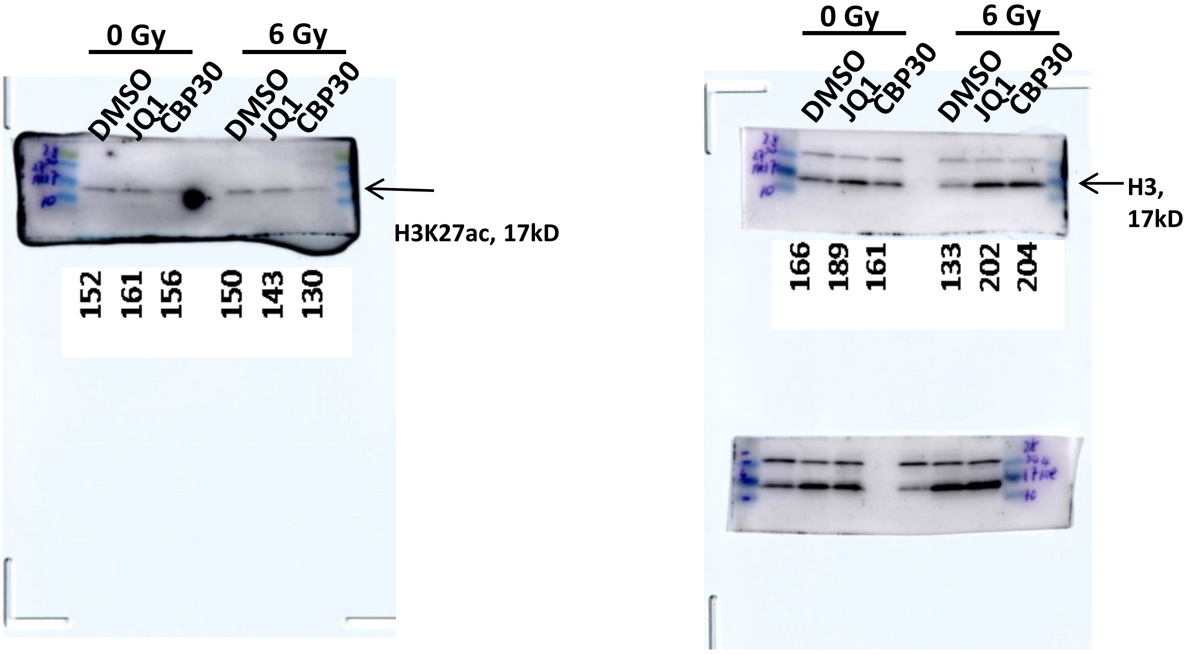

Figure 5A, image for statistic

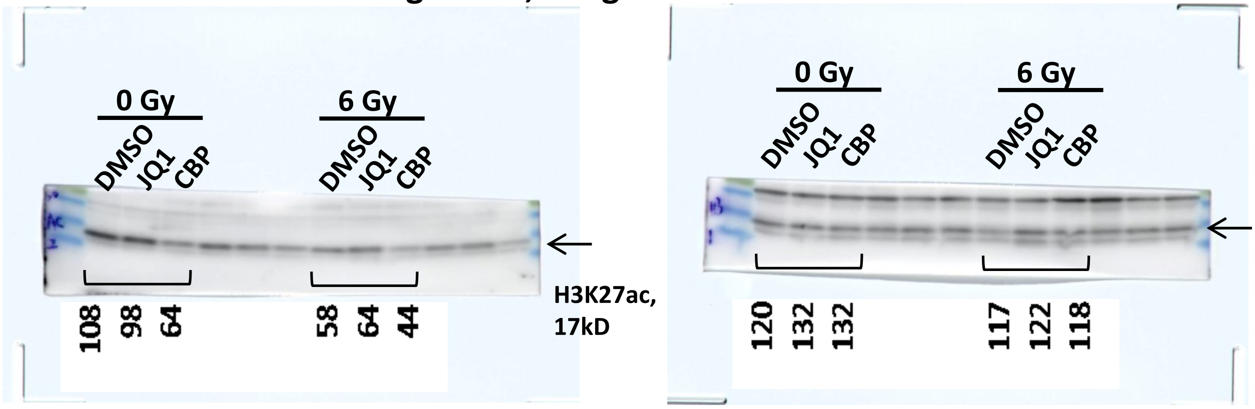

Figure S11. Original Western blot images for Figure S1A.

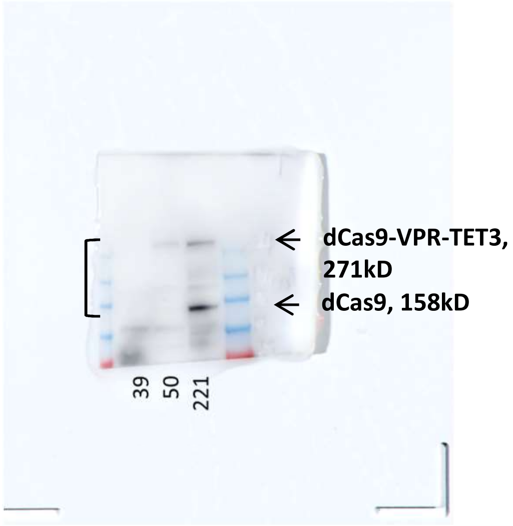

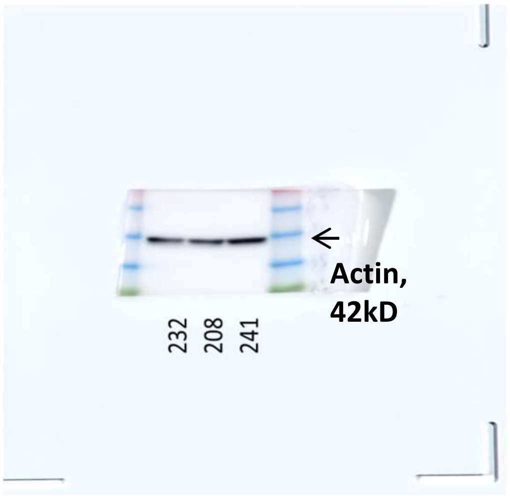

Supplement: Supplementary file 1 [file cancers-13-02455-s001.zip › mergeSupFig.pdf]
